# *In situ* Neutron Diffraction during Casting: Determination of Rigidity Point in Grain Refined Al-Cu Alloys

**DOI:** 10.3390/ma7021165

**Published:** 2014-02-12

**Authors:** Jean-Marie Drezet, Bastien Mireux, Zoltan Szaraz, Thilo Pirling

**Affiliations:** 1 Computational Materials Laboratory, Ecole Polytechnique Federale Lausanne, station 12, Lausanne CH-1015, Switzerland; E-Mail: bastien.mireux@epfl.ch; 2 Institut Laue Langevin, Salsa instrument, Grenoble F-38042, France; E-Mails: szaraz@ill.fr (Z.S.); pirling@ill.fr (T.P.)

**Keywords:** solidification, coalescence, rigidity temperature, hot tearing

## Abstract

The rigidity temperature of a solidifying alloy is the temperature at which the solid plus liquid phases are sufficiently coalesced to transmit long range tensile strains and stresses. It determines the point at which thermally induced deformations start to generate internal stresses in a casting. As such, it is a key parameter in numerical modelling of solidification processes and in studying casting defects such as solidification cracking. This temperature has been determined in Al-Cu alloys using *in situ* neutron diffraction during casting in a dog bone shaped mould. In such a setup, the thermal contraction of the solidifying material is constrained and stresses develop at a hot spot that is irradiated by neutrons. Diffraction peaks are recorded every 11 s using a large detector, and their evolution allows for the determination of the rigidity temperatures. We measured rigidity temperatures equal to 557 °C and 548 °C, depending on cooling rate, for a grain refined Al-13 wt% Cu alloy. At high cooling rate, rigidity is reached during the formation of the eutectic phase and the solid phase is not sufficiently coalesced, *i.e.*, strong enough, to avoid hot tear formation.

## 1. Introduction

Coalescence is a key transition in solidification of metallic alloys. It corresponds to the formation of solid bridges between grains when both solid and liquid phases are percolated [1]. Coalescence starts at the coherency point when the grains begin to touch each other, but are unable to sustain any tensile loads. Rappaz *et al.* [2] have used the concept of disjoining-pressure used in fluid dynamics to establish a theoretical framework for the coalescence of primary phase dendritic arms within a single grain or at grain boundaries. For pure substances, approaching planar liquid/solid interfaces coalesce to a grain boundary at an undercooling Δ*T*_b_ given by:

$$\Delta T_{b}=\frac{\gamma_{\mathrm{gb}}-2\gamma_{\mathrm{sl}}}{\Delta S_{f}}\frac{1}{\delta }$$ (1)

where δ is the thickness of the solid-liquid interface, γ_gb_ the grain boundary energy, γ_sl_ the solid/liquid interfacial energy and Δ*S*_f_ the entropy of fusion. The quantity γ_gb_ − 2γ_sl_ depends on the grain misorientation. When it is positive, the two liquid/solid interfaces are “repulsive.” In this case, a stable liquid film between adjacent dendrite arms located across such grain boundaries can remain until the undercooling exceeds Δ*T*_b_. For alloys, coalescence is also influenced by the concentration of the liquid films. The temperature and concentration of the liquid films must reach a coalescence line parallel to, but Δ*T*_b_ below, the liquidus line before coalescence can occur. At the macroscopic level, *i.e.*, at the level of many randomly oriented grains, coalescence must be considered as a transition taking place between coherency (first contact between the grains) and rigidity (ability to transmit tensile strains and stresses). If coalescence between some grains is retarded, *i.e.*, finishes at lower temperatures, the mushy structure becomes particularly sensitive to hot tearing or solidification cracking [3]. This defect is a spontaneous failure of semi-solid metallic alloys that results in an intergranular fracture profile. It appears during casting near the end of solidification, especially in low solute content alloys where straining becomes localized and local liquid permeability is very low. At the macroscopic level, coalescence ends at the rigidity point, also called mechanical coherency, when the structure is able to sustain substantial tensile strains and stresses, *i.e.*, when the solid phase is sufficiently percolated. The rigidity temperature is important as it determines the very instant macroscopic stresses start to build up owing to thermally induced deformations [4].

The mechanical behavior of alloys in the mushy state is being intensively studied using X-ray microtomography. For example, recent *in situ* tensile test experiments have been performed on mushy alloys at the X-ray synchrotron of the European Synchrotron Radiation Facility (ESRF) by Terzi *et al.* [5,6]. The major drawbacks of such *in situ* tensile tests are that they are carried out in isothermal conditions, and not during cooling, since XR imaging requires time for image acquisition and liquid state cannot be investigated owing to rotations required for imaging [7]. Moreover, the exact mechanical loading within the material is not known as the deformation localized in the hottest part of the specimen is not measured. These tests are presently limited to Al-Cu alloys [8] in order to get a good absorption contrast, and the alloy is tested after heating to the correct temperature within the solidification interval and not during solidification from the fully liquid state. The last point is particularly detrimental as it is well known that solidifying microstructures are different from those obtained after heating owing to dendrite coarsening and redistribution of solute elements [9]. Fallet *et al.* [10] studied the influence of a barium addition to Al-Cu alloys on the morphology of liquid films in the mushy zone and showed that barium improves wetting of the solid phase by the liquid (decrease of γ_sl_ in Equation (1)) and thus delays coalescence of the grains. They carried out tensile and shear tests during solidification and observed that the fracture stress in tension is affected by the presence of Ba at the very end of solidification. This is due to the delayed coalescence of Ba-treated alloys for which liquid films embrittle the alloy in tension up to very high solid fractions.

To our knowledge, *in situ* studies of stress accumulation in solidifying metals have been very limited, with only one unpublished study reported on cast aluminium composite structures. This study has shown that neutron diffraction at high flux sources is particularly well suited for such investigations as it would allow determining the very moment when strains and stresses appear in the mushy alloy. Although badly assessed, the rigidity temperature is a key parameter in solidification. It highly varies from one alloy to another and depends on both the cooling rate and the degree of grain refinement, *i.e.*, on the grain structure such as equiaxed, columnar, globulitic or globular. It is an important input data in numerical modeling of as-cast residual stresses in billets [11,12] and in rolling sheet ingots [13] as it dictates the temperature below which thermal strains start to occur.

The present paper reports on the use of *in situ* neutron diffraction during solidification. Rigidity temperature has been measured at the Salsa neutron diffractometer [14] in Al-Cu alloys under various cooling regimes in a dog bone shaped mould where solidification and tensile straining are concomitant.

## 2. In situ Neutron Diffraction during Casting

**Figure 1.**
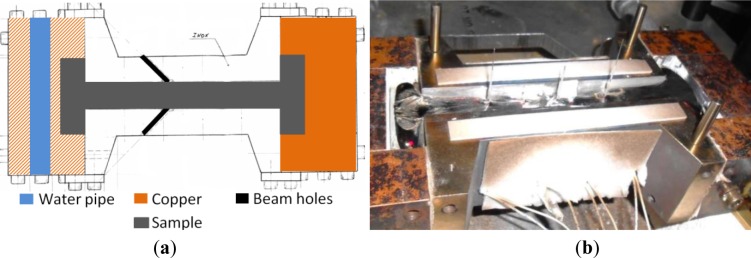
(**a**) Mould design; (**b**) Dog bone shaped casting.

Al-13 wt% Cu alloys were prepared by melting Al-4.43 wt% Cu alloy and pure copper in an alumina crucible at 750 °C. They were grain refined with 0.4 wt% Al-TiB_2_ master alloy and cast in dog bone shaped moulds. The setup used to study the hot tearing susceptibility of new alloys is presented in Figure 1. Both extremities of the casting were cooled using air or water. A hot spot forms at the center of the casting and its axial contraction is prevented by the steel mould surrounding the central part. The thermal contraction of the solidifying material is constrained and stresses develop at the hot spot, and may lead to hot cracking. The major advantage of this configuration is that solidification and tensile straining are concomitant. It is possible to control the amount of constraint on the hot spot by modulating the air or water cooling of the mould extremities and by preheating the mould.

In order to measure the accumulation of strain and thus of stress during casting, the lattice spacing was measured *in situ* at the hot spot location using neutron diffraction. Small holes were machined in the steel mould to provide unimpeded access to the sample for the neutron beam. Insulating alumina muffles were used to avoid liquid metal leakage. Type K thermocouples placed at precise locations within the casting allowed linking the temperature within the gauge volume with the formation and shift of the (311) Al-Cu solid solution diffraction peak during solidification, which permitted the measurement of rigidity temperature. The experiments were carried out with a gauge volume of 2 × 2 × 15 mm^3^, using an open slit along gravity, and a casting cross section of 15 × 25 mm^2^. A large ion chamber neutron detector (2D position sensitive, 260 × 260 mm^2^ active area with a 1 mm resolution, 256 × 256 channels) allowed us to acquire exploitable peaks within 5 s. With roughly 6 s between acquisitions, one diffraction peak was recorded roughly every 11 sec during casting. As the alloy was grain refined, no texture appeared.

## 3. Results and Discussion

**Figure 2.**
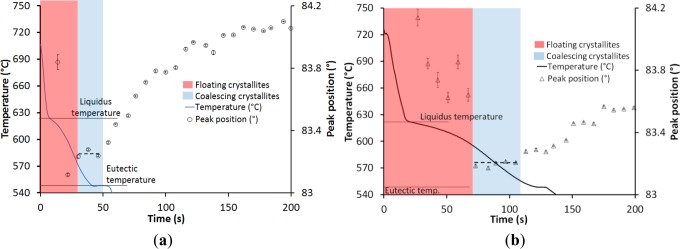
Temperature in gauge volume and peak position in (**a**) Sample 1; (**b**) Sample 2.

**Figure 3.**
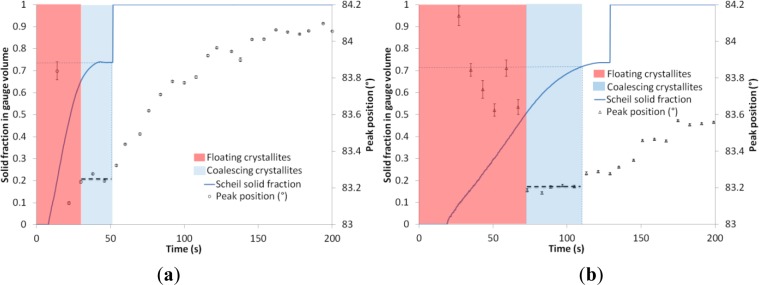
Solid volume fraction in gauge volume and peak position in (**a**) Sample 1; (**b**) Sample 2.

Figure 2 shows the evolution of both the temperature at the hot spot and the diffraction angle of the Al-Cu solid solution for two samples cast at two different cooling rates. Sample 1 exhibited some hot tears at the hot spot, but was not fully fractured, whereas Sample 2 was sound, *i.e.*, free of hot tears. Solidification starts at 623 °C with no undercooling, as the alloys are grain refined. This liquidus temperature corresponds to a 13 wt% Cu content. Solidification ends with the formation of the eutectic phase, 26% in volume fraction. Copper is known to diffuse very slowly in solid aluminium. Thus, the volume fraction of solid is calculated using the Scheil’s model. Figure 3 shows both the evolution of the solid volume fraction at the hot spot and the diffraction angle for the two samples.

The earliest peaks have erratic angle values with relative angular error higher than 10%. They correspond to the diffraction of neutrons by crystallites floating freely in the liquid phase. The peaks then exhibit better quality with a relative angular error lower than 2% and their positions stabilize over three peaks in Sample 1 cast rapidly (40 s solidification time) and five peaks in Sample 2 cast with a lower cooling rate (100 s solidification time) when grains touch each other (solid percolation) and start to coalesce. The rotation and displacement of the grains are constrained, but tensile stress still can’t be transmitted. The temperature at this instant is considered as the coherency temperature and corresponds to the beginning of the coalescence transition. The exact determination of this temperature is uneasy as very few peaks are present. Nevertheless, it seems that the coherency temperature is around 584 °C for Sample 1 and 592 °C for Sample 2. After a certain time the peak position starts to increase. This denotes the instant when the structure reaches its rigidity point and becomes able to sustain tensile straining. Rigidity temperature corresponds to the eutectic temperature, 548 °C, in Sample 1, and to 557 °C (solid volume fraction around 71%) in Sample 2. Coalescence is presented schematically in Figures 2 and 3. During the transition, peak positions and thus lattice spacing are relatively constant. Small variations are due to thermal contraction and copper segregation. Coalescence starting at the coherency temperature and ending at the rigidity temperature lasts about 40 s in Sample 1 and 100 s in Sample 2.

**Figure 4.**
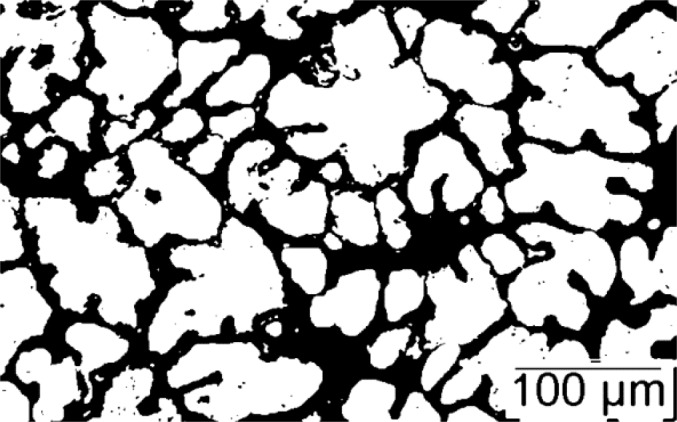
Binarized micrograph of Sample 1.

**Figure 5.**
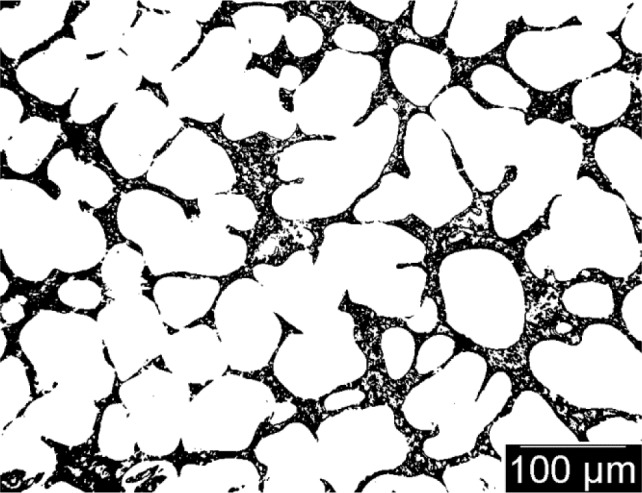
Binarized micrograph of Sample 2.

Optical micrographs, presented in Figures 4 and 5 for both samples, show the presence of small grains (~100 microns) surrounded by a fine intergranular eutectic structure. In Sample 1, the grains are dendritic with secondary arm spacing of about 20 μm. In Sample 2, the grains are more globular and it is difficult to determine the secondary arm spacing. The binarized images reveal also that the grains are percolated, *i.e.*, bridged together in Sample 2, whereas the eutectic phase is not percolated and isolated in between the grains. In contrast, in Sample 1 where hot tears appeared, the grains are not percolated, but the eutectic phase is. Microporosity is also found in this sample close to the surface of the casting.

These findings agree with previous studies on hot tearing. Terzi *et al.* [5] and Sistaninia *et al.* [15] reported that mechanical instabilities of micropores at the surface of the sample lead to the formation of hot tears. Hot tearing at the hot spot of Sample 1 is explained by a delayed coalescence that starts at a lower coherency temperature and ends with the eutectic formation. This situation is similar to the Barium treated alloys studied by Fallet *et al.* [10].

In order to determine strains, strain rates and stresses once rigidity is reached, the experiments need to be conducted in a free-to-contract configuration in order to measure the evolution of the lattice parameter with decreasing temperature. The difference in peak positions between the constrained and free-to-contract configurations will allow us to calculate the stress and strain build up in sound samples. These quantities will then be used to determine the alloy as-cast rheology.

## 4. Conclusions

This study shows that neutron diffraction can be used to determine coherency and rigidity points, *i.e.*, coalescence transition, in solidifying aluminium alloys. Using a large detector is a prerequisite to obtain enough diffraction peaks in the semi-solid state. Rigidity temperatures of 557 °C and 548 °C, depending on cooling rate, have been determined for the grain refined Al-13 wt% Cu alloy. At high cooling rate, rigidity is reached during the formation of the eutectic phase and the solid phase is not sufficiently coalesced, *i.e.*, strong enough, to avoid hot tear formation. The next step is to repeat the experiments in a free-to-contract configuration, in order to measure the lattice parameter with decreasing temperature and calculate the stress and strain build up in sound samples. With a better time resolution, the determination of coherency and rigidity points should be feasible in experimental conditions closer to the industrial ones, *i.e.*, at higher cooling rates.
